# A requirement for the use of supplementary oxygen to guide medical treatment decisions may introduce bias against non-white individuals

**DOI:** 10.1183/13993003.02320-2023

**Published:** 2024-06-06

**Authors:** Colin J. Crooks, Joe West, Joanne R. Morling, Mark Simmonds, Irene Juurlink, Steve Briggs, Simon Cruickshank, Susan Hammond-Pears, Dominick Shaw, Timothy R. Card, Andrew W. Fogarty

**Affiliations:** 1 Nottingham Digestive Diseases Centre, School of Medicine, University of Nottingham, Nottingham, UK; 2 NIHR Nottingham Biomedical Research Centre (BRC), Nottingham University Hospitals NHS Trust and the University of Nottingham, Nottingham, UK; 3 Nottingham University Hospitals NHS Trust, Nottingham, UK; 4 Lifespan and Population Health, School of Medicine, University of Nottingham, Nottingham, UK; 5 East Midlands Academic Health Science Network, University of Nottingham, Nottingham, UK; 6 NIHR Leicester Respiratory Biomedical Research Centre and University of Leicester, Leicester, UK

## Abstract

In England and Wales there was excess mortality from coronavirus disease 2019 (COVID-19) infection in individuals whose forebears originated outside the UK, often in Africa or South East Asia [1]. The reasons for this are still unclear. We suggest that the use of oxygen saturations derived from pulse oximetry to guide treatment may have been a contributory factor.

## Shareable abstract

**The use of supplementary oxygen to guide medical treatment decisions in guidelines may introduce bias against non-white individuals** https://bit.ly/4b8AFfw

*To the Editor*:

In England and Wales there was excess mortality from coronavirus disease 2019 (COVID-19) infection in individuals whose forebears originated outside the UK, often in Africa or South East Asia [1]. The reasons for this are still unclear. We suggest that the use of oxygen saturations derived from pulse oximetry to guide treatment may have been a contributory factor.

Pulse oximeters provide a noninvasive measure of blood oxygen saturation [2], and were commonly used during the COVID-19 pandemic. However, pulse oximetry is less accurate in patients with non-white skin [3, 4], leading to risks of misleadingly higher estimates of oxygen saturation. This could delay starting oxygen supplementation in patients with non-white skin. Given that guidelines for managing COVID-19 pneumonia state that corticosteroids should be prescribed to patients receiving supplementary oxygen [5, 6], any racial bias from pulse oximeters in patients with non-white skin also has the potential to delay prescription of corticosteroids, potentially deferring an intervention that reduces mortality by 18% [7].

We hypothesised that in patients admitted with COVID-19 infection, those of non-white ethnicity would have more severe respiratory failure, as defined as a higher respiratory rate compared to those of white ethnicity, when their treatment was increased by starting supplementary oxygen therapy.

We conducted a cohort study using routinely collected electronic data for patients admitted to Nottingham University Hospitals NHS trust between 1 February 2020 and 31 December 2021 with COVID-19 infection. There were no data on oxygen use prior to arrival at hospital, *e.g.* in an ambulance. Hence, there were two complementary analyses stratified by ethnic group. Firstly, the initial set of observations associated with a first use of supplementary oxygen during the hospital admission. Secondly, the last set of observations documented with no supplementary oxygen before receiving supplementary oxygen therapy in hospital.

A linear model predicting each measurement by ethnicity was fitted, adjusted for age (as a linear and a categorical variable ≤50 years, 51–60 years, 61–70 years, 71–80 years and >80 years) and sex, for both analyses. All analyses were performed using version 4.2.2 of the R programming language.

Approval for this work was granted *via* an NUH Clinical Effectiveness Team audit (reference: 21-294C) and IRAS (REC: 20/WM/0142, project ID: 282490, amendment number SA02 20/07/21).

Data were available from a total population of 7930 eligible adults who had a median age of 69 years. 5822 (73%) of these individuals were classified as being of white ethnicity. When initially recorded as starting supplementary oxygen, the median blood oxygen saturation derived from pulse oximetry was 96% for each of the white, black and South East Asian ethnic groups in the 7810 patients with available data.

In the initial analysis that included patients at the first recorded use of supplementary oxygen during an admission to hospital, individuals with an ethnicity classified as either black or South East Asian had a higher respiratory rate of +1.6 breaths·min^−1^ (95% CI +0.9 to +2.3) and +1.3 (95% CI +0.6 to +1.9), respectively, than those classified as white (table 1), after adjustment for age and sex. In the second analysis of patients who were documented as not initially requiring supplementary oxygen in hospital, before treatment was escalated to the prescription of supplementary oxygen, the adjusted association with black ethnicity increased to +2.7 breaths·min^−1^ (95% CI +0.2 to +5.2) compared to those of white ethnicity (table 1), although the association with South East Asian ethnicity did not persist.

**TABLE 1:** Respiratory rate of patients with COVID-19 infection at time of commencing supplementary oxygen stratified by reported ethnicity

	Subjects n	Respiratory rate at first recorded use of supplementary oxygen during admission	p-value	Subjects n	Respiratory rate on air prior to initiation of supplementary oxygen during admission	p-value
**Age** **(years)**	7930	0.00 (−0.01 to 0.01)	0.609	579	−0.01 (−0.04 to +0.01)	0.277
**Ethnicity**						
White	5822	Reference		414	Reference	
Asian	373	+1.25 (+0.60 to +1.89)	**<0.001**	22	+0.51 (−1.94 to +2.97)	0.680
Black	298	+1.62 (+0.90 to +2.33)	**<0.001**	21	+2.70 (+0.21 to +5.18)	**0.033**
Other	171	+1.64 (+0.70 to +2.57)	**0.001**	18	+3.86 (+1.16 to +6.55)	**0.005**
Unknown	1266	+0.69 (+0.31 to +1.06)	**<0.001**	104	+0.15 (−1.08 to +1.37)	0.816
**Sex**						
Male	4202	Reference		300	Reference	
Female	3728	−0.46 (−0.73 to −0.18)	**0.001**	279	+0.19 (−0.73 to +1.12)	0.678

Data on respiratory rate are adjusted differences in rate with 95% confidence intervals, in breaths·min^−1^. Analysis using multivariate linear regression. Significant p-values are presented in bold type.

This is the first analysis to demonstrate that COVID-19 patients of black ethnicity had increased respiratory rate prior to the initiation of supplementary oxygen compared to patients with white ethnicity. The most likely explanation for this observation is the differential error of oxygen saturation when measured by pulse oximeters in people with black ethnicity.

The strengths of this analysis are the collection of data from all patients with a diagnosis of COVID-19 infection admitted to a busy UK teaching hospital. Data were recorded on ethnicity during the routine admission procedure. Data were collected on respiratory rate, oxygen saturation as measured by pulse oximetry and the presence or absence of supplementary oxygen as part of the routine clinical observations, and these data were electronically archived. A strength of the analysis is the availability of data on the patients who did not receive supplementary oxygen on arrival to hospital, but then required it.

Routinely collected data on ethnic group as categorised by skin pigmentation may be susceptible to measurement error, however, it is likely that individuals labelled as white, South East Asian and black ethnicity are from broadly recognisable phenotypes. Respiratory rate is often manually measured by healthcare staff [8], and this can also introduce measurement error that may reduce the precision and size of the differences observed. However, this would likely decrease any differences observed as a result of regression dilution bias [9].

The problem of differential error of oxygen saturation as measured by pulse oximetry in individuals with black skin has been known for at least three decades [10], and subsequently reported in larger populations in the USA [3] and in patients from our institution [4]. These data are consistent with our prior observation that at the time of transfer to intensive care, those patients of black ethnicity had substantially more severe respiratory failure, with increased respiratory rates and decreased oxygen saturation as measured by arterial blood gases, yet similar oxygen saturation as measured by pulse oximeters [11]; again, this would be consistent with the hypothesis that differential error of oxygen saturation as measured by pulse oximeters is associated with delays in the escalation of treatment in patients with non-white skin compared to those with white skin.

These data provide further evidence that differential measurement error of oxygen saturation in patients with non-white skin may contribute to the excess mortality from COVID-19 infections [1]. As such, it highlights the need for further analysis of other large datasets, as these findings could have important implications for clinical decision-making. Datasets that have access to concurrent electronic prescribing data would be particularly useful, as these could allow evaluation of the timing of the initiation of therapies such as corticosteroids with the physiological measurements of disease severity. These potential analyses could also explore other key data elements in detail, such as patient clinical characteristics, medical management strategies and outcome data, that we were unable to analyse in our dataset.

Pulse oximeters are used to assess patients in primary care, ambulances, emergency departments and hospitals, both as part of both direct clinical care and also the early warning score safety net surveillance system [12], as well as in virtual wards at home after discharge from hospital [13]. This problem is compounded if the initiation of effective treatments that reduce mortality, such as the prescription of corticosteroids in COVID-19 infection, is linked to disease severity as defined by the requirement for supplementary oxygen, which is a decision that is often based on pulse oximetry measurements.

When designing guidelines in respiratory disease, awareness that the differential measurement error of oxygen saturation (as measured by pulse oximeters) across different ethnic groups may modify thresholds for starting oxygen therapy is an important consideration, particularly if the use of supplementary oxygen is linked to starting disease modifying treatments. This may be particularly important in the context of future pandemics.

## Shareable PDF

10.1183/13993003.02320-2023.Shareable1 This one-page PDF can be shared freely online.
Shareable PDF ERJ-02320-2023.Shareable
