# Supplementary material for: A requirement for the use of supplementary oxygen to guide medical treatment decisions may introduce bias against non-white individuals
Source: Eur Respir J. 2024 Jun 6;63(6):2302320. doi: 10.1183/13993003.02320-2023 (PMC11154750; doi:10.1183/13993003.02320-2023)
Supplement: Supplementary file 1 [file ERJ-02320-2023.Shareable.pdf]

SHAREABLE PDF

A requirement for the use of supplementary oxygen to guide medical treatment decisions may introduce bias against non-white individuals

Colin J. Crooks [1,2,3], Joe West [2,3,4,5], Joanne R. Morling[2,3,4], Mark Simmonds[3], Irene Juurlink [3], Steve Briggs [3], Simon Cruickshank [3], Susan Hammond-Pears [3,5], Dominick Shaw [6], Timothy R. Card [2,3,4] and Andrew W. Fogarty[2,3,4]

[1]Nottingham Digestive Diseases Centre, School of Medicine, University of Nottingham, Nottingham, UK. [2]NIHR Nottingham Biomedical Research Centre (BRC), Nottingham University Hospitals NHS Trust and the University of Nottingham, Nottingham, UK. [3]Nottingham University Hospitals NHS Trust, Nottingham, UK. [4]Lifespan and Population Health, School of Medicine, University of Nottingham, Nottingham, UK. [5]East Midlands Academic Health Science Network, University of Nottingham, Nottingham, UK. [6]NIHR Leicester Respiratory Biomedical Research Centre and University of Leicester, Leicester, UK.

Corresponding author: Andrew W. Fogarty (andrew.fogarty@nottingham.ac.uk)

Check for updates

Shareable abstract (@ERSpublications)
The use of supplementary oxygen to guide medical treatment decisions in guidelines may introduce bias against non-white individuals https://bit.ly/4b8AFfw

Cite this article as: Crooks CJ, West J, Morling JR, *et al*. A requirement for the use of supplementary oxygen to guide medical treatment decisions may introduce bias against non-white individuals. *Eur Respir J* 2024; 63: 2302320 [DOI: 10.1183/13993003.02320-2023].

This extracted version can be shared freely online.

Copyright ©The authors 2024.

This version is distributed under the terms of the Creative Commons Attribution Licence 4.0.

Received: 25 Dec 2023
Accepted: 2 May 2024

To the Editor:

In England and Wales there was excess mortality from coronavirus disease 2019 (COVID-19) infection in individuals whose forebears originated outside the UK, often in Africa or South East Asia [1]. The reasons for this are still unclear. We suggest that the use of oxygen saturations derived from pulse oximetry to guide treatment may have been a contributory factor.
